# Vein-first vs artery-first surgical technique for lobectomy of non-small cell lung cancer: A protocol for systematic review and meta-analysis

**DOI:** 10.1097/MD.0000000000020768

**Published:** 2020-06-26

**Authors:** Lei Gao, Zhimin Shen, Hui Xu, Fei Luo, Peipei Zhang, Tianci Chai, Sui Chen, Mingqiang Kang

**Affiliations:** Department of Thoracic Surgery, Fujian Medical University Union Hospital, Fuzhou, China.

**Keywords:** artery-first surgical technique, lobectomy, non-small cell lung cancer, vein-first surgical technique

## Abstract

**Background:**

The operation of lung cancer may squeeze the tumor and further promote the spread of tumor cells to the circulation, which may be one of the reasons for the metastasis and recurrence of lung cancer. The potential risk of tumor cell dissemination can theoretically be minimized if the effluent veins were ligated first (via the vein-first [V-first] technique), instead of having the artery ligated first (via the artery-first [A-first] technique). However, this technical concept has not yet been widely accepted as a standard of surgical oncology in current guidelines owing to a lack of sufficient evidence. This systematic review and meta-analysis will be performed to determine which technique during lobectomy will achieve longer patient survival and be more beneficial for patients.

**Methods:**

We will search PubMed, Web of Science, EMBASE, Cancerlit, the Cochrane Central Register of Controlled Trials, and Google Scholar databases for relevant clinical trials published in any language before October 1, 2020. Randomized controlled trials (RCTs), quasi-RCTs, propensity score-matched comparative studies, and prospective cohort studies of interest, published or unpublished, that meet the inclusion criteria will be included. Subgroup analysis of the type of operation, tumor pathological stage, and ethnicity will be performed. INPLASY registration number: INPLASY202050060.

**Results:**

The results of this study will be published in a peer-reviewed journal.

**Conclusion:**

As far as we know, this study will be the first meta-analysis to compare the efficacy of the vein-first and artery-first surgical technique of lobectomy for patients diagnosed with resectable non-small cell lung cancer. Due to the nature of the disease and intervention methods, randomized controlled trials may be inadequate, and we will carefully consider inclusion in high-quality, non-randomized controlled trials, but this may result in high heterogeneity and affect the reliability of the results.

## 1 Introduction

Surgery is the preferred treatment for many solid tumors, such as non-small cell lung cancer (NSCLC), esophageal cancer, and hepatocellular carcinoma. However, even after radical resection, about 50% of patients may experience local recurrence or distant metastasis within 3 years.^[1–4]^

Numerous studies have demonstrated that surgical manipulation could promote the dissemination of tumor cells into the circulation.^[5–9]^ The operation of lung cancer may squeeze the tumor and further promote the spread of tumor cells to the circulation, which may be one of the reasons for the metastasis and recurrence of lung cancer. As reported by McCulloch et al, tumor cells can be detected in effluent venous blood during surgery.^[10]^ In addition, vascular invasion within the tumor is also common in lung cancer, which might be responsible for the high incidence of hematogenous spread of tumor cells.^[11–13]^ A surgical technique that may prevent the dissemination of tumor cells into the bloodstream is to ligate the effluent veins first during surgery.^[14]^

The potential risk of tumor cell dissemination can theoretically be minimized if the effluent veins were ligated first (via the vein-first [V-first] technique), instead of having the artery ligated first (via the artery-first [A-first] technique). However, this technical concept has not yet been widely accepted as a standard of surgical oncology in current guidelines owing to a lack of sufficient evidence. This systematic review and meta-analysis will be performed to determine which technique during lobectomy will achieve longer patient survival and be more beneficial for patients with resectable NSCLC.

## 2 Objective

We will conduct a systematic review and meta-analysis to estimate the efficacy and safety of the vein-first and artery-first surgical technique of lobectomy for patients diagnosed with resectable NSCLC.

## 3 Methods

This protocol adheres to the Preferred Reporting Items for Systematic Review and Meta-Analysis Protocols (PRISMA-P) statement.^[15]^ The results of this systematic review and meta-analysis will be published with reference to the Preferred Reporting Items for Systematic Review and Meta-Analysis (PRISMA) guidelines.^[15]^

Patient and public involvement: This study will be based on published or unpublished studies, and records and will not involve patients or the public directly.

### 3.1 Eligibility criteria

#### 3.1.1 Types of studies

Randomized controlled trials (RCTs), quasi-RCTs, propensity score matched comparative studies and prospective cohort studies of interest, published or unpublished, will be included. These should be completed, and the efficacy and safety of the vein-first vs artery-first surgical technique of lobectomy for patients diagnosed with resectable NSCLC.

#### 3.1.2 Types of participants

The participants will be patients diagnosed with resectable, pathologically confirmed NSCLC who were treated with lobectomy, and there will be no restrictions on sex, ethnicity, economic status, or education.

#### 3.1.3 Types of interventions

All types of vein-first or artery-first surgical technique of lobectomy for patients diagnosed with resectable NSCLC will be studied.

#### 3.1.4 Types of outcome measures

##### 3.1.4.1 Primary outcomes

The primary outcome will be overall survival of patients with respectable NSCLC after surgery.

##### 3.1.4.2 Secondary outcomes

We will evaluate the 5-year survival, recurrence-free survival, and median survival rates as well as the quality of life and complication rate of patients with resectable NSCLC after surgery.

### 3.2 Information sources

Two reviewers (LG and SZM) will search PubMed, Web of Science, Cancerlit, Embase, Cochrane Central Register of Controlled Trials, and Google Scholar databases for relevant trials published before October 1, 2020, without any language restrictions.

### 3.3 Search strategy

The subject terms and keywords corresponding to medical subject heading (MeSH) terms will be used to search for eligible trials in the databases as mentioned above with no language restrictions. Search strategies in PubMed are shown in Table 1.

**Table 1:**
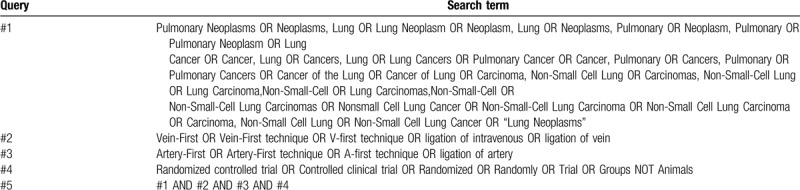
PubMed search strategies.

### 3.4 Data collection and analysis

We will adopt the methods described in the Cochrane Handbook for Systematic Reviews of Interventions to pool the evidence.^[16]^

#### 3.4.1 Study selection

Two authors (LG and SZM) will independently screen each title and abstract of all the papers searched, and the trials that do not meet the inclusion criteria described in this protocol will be excluded. The full text of all the possibly eligible trials will be screened independently and in duplicate by the two authors. Trials that are irrelevant or do not meet the inclusion criteria will be excluded. Trials that meet the inclusion criteria and excluded studies along with the reasons for their exclusion will be documented by the 2 authors (LG and SZM). If there is a disagreement between the 2 authors, we will come to a resolution by discussing it with the third author (MQK). If necessary, we will consult the fourth author (KMQ) to resolve the disagreement. The selection process will be shown in a PRISMA flow chart in detail.

#### 3.4.2 Data extraction and management

We will extract the following data from the included trials.

- Study characteristics: author, publication date, country, study design, randomization, periods of data collection, follow-up duration, withdrawals, and overall duration of study.
- Population characteristics: age, sex, pathology diagnosis, tumor stage, pathologic tumor size, performance status, ethnicity, history of smoking, and inclusion criteria.
- Interventions: type of operation, number of lymph nodes retrieved, extent of resection, duration of operation, bleeding, and postoperative adjuvant therapy.
- Outcomes: overall survival, 5-year survival, recurrence-free survival, median survival, length of stay, length of ICU stay, quality of life, complications, and adverse events.

We will use the pre-designed table to record the data extracted from the included trials. If relevant data from the trials are lost or unclear, we will consult the author via email before determining whether the study is to be included.

### 3.5 Assessment of risk of bias

The Cochrane Handbook for Systematic Reviews of Interventions will be used to assess the risk of bias of each trial included. The two authors (LG and SZM) will evaluate the risk of bias based on the following domains: random sequence generation (selection bias), allocation concealment (selection bias), blinding of participants and personnel (performance bias), blinding of outcome assessment (detection bias), incomplete outcome data (attrition bias), selective outcome reporting (reporting bias), and other biases.^[17]^ The risk of bias in each domain will be assessed as high, low, or uncertain, and the results of the evaluation will be shown on the risk of bias graph. EPOC guidelines will be used to assess the risks of the non-randomized controlled trials included.^[18]^

### 3.6 Data analysis

We will use Review Manager and Stata software to synthesize the data extracted. If the data extracted from the included studies are evaluated as highly homogeneous, we will use them to conduct a meta-analysis for the purpose of obtaining a clinically meaningful result. To carry out a standard meta-analysis, we will use the Chi^2^ and I^2^ statistical tests to evaluate statistical heterogeneity among the studies. If there is high heterogeneity (*P* < .1 or I2 statistic >50%), we will use the DerSimonian and Laird random effect model to analyze the extracted data. Because high heterogeneity may be caused by different types of tumors and different stages of tumors diagnosed by pathology and different means of adjuvant therapy may be used after the operation, we will perform a subgroup analysis of the types of tumors (esophageal squamous cell carcinoma and esophageal adenocarcinoma), the pathological stages of the tumors, and the means of adjuvant therapy after the operation (types of chemotherapeutic drugs and whether or not radiotherapy is accepted). Otherwise, we will adopt a fixed-effect model to analyze the data. We will adopt the Mantel-Haenszel method to pool the binary data, and the results will be reported in the form of relative risk (RR) with a 95% confidence interval. An inverse variance analysis method will be used to pool the continuous data, and the results will be reported in the form of a standardized mean difference (SMD) with a 95% confidence interval.

#### 3.6.1 Subgroup analysis

If there is substantial heterogeneity and if the available data are sufficient, we will perform subgroup analysis to search for potential origins of heterogeneity. If the extracted data are enough, we will conduct subgroup analysis of the type of operation, type of tumor, tumor stage, age, and postoperative adjuvant treatment.

#### 3.6.2 Sensitivity analysis

We will conduct a sensitivity analysis to evaluate the robustness and reliability of the aggregation results by eliminating trials with a high bias risk. If a reporting bias exists, we will use the methods of fill and trim to analyze for publication bias.^[19]^

### 3.7 Publication bias

Funnel charts and Egger test will be adopted to assess for publication bias if there are no less than ten eligible trials. If reporting bias is suspected in a trial, we will contact the corresponding author via email to determine whether there are additional outcome data that were not reported.

### 3.8 Evidence evaluation

We will classify the quality of all the evidence into four levels (high, medium, low and very low) in accordance with the criteria of grading of recommendations, assessment, development and evaluation (study limitations, imprecision, publication bias, indirectness bias, and effect consistency).^[20]^

## 4 Discussion

V-first technology has been proved to reduce the diffusion of tumor cells into the circulation during the operation, which is beneficial to the potential survival of patients with NSCLC.^[21]^ These better results may be due to the reduction of the number of tumor lobes and the avoidance of squeezing tumor cells into circulation during surgery. In the V-first procedure, the pulmonary vein of the shallowest hilus was dissected and cut off, and then the branches of bronchus and pulmonary artery were dissected. The operation avoids repeated compression and tumor bearing leaf turnover. In addition, once the draining vein is blocked, tumor cells are unlikely to enter the bloodstream.^[22,23]^

The V-first technique of pneumonectomy has been proposed by some surgeons. However, some surgeons still maintain that continuous pulmonary artery inflow into the lobes of the ligated veins can result in a loss of intravascular volume. On the other hand, A-first may have the advantage of preventing unnecessary blood loss of excised lobes.^[24,25]^ The potential risk of tumor cell dissemination can theoretically be minimized if the effluent veins were ligated first, instead of having the artery ligated first. However, this technical concept has not yet been widely accepted as a standard of surgical oncology in current guidelines owing to a lack of sufficient evidence. This systematic review and meta-analysis will be performed to determine which technique during lobectomy will achieve longer patient survival and be more beneficial for patients with resectable NSCLC.

As far as we know, this study will be the first systematic review and meta-analysis to compare the efficacy and outcome of the 2 different surgical techniques to determine which is more likely to benefit patients with NSCLC and to provide a basis for clinicians to develop optimal treatment strategies for patients.

## Author contributions

Mingqiang Kang and Sui Chen is the guarantor of the article. Lei Gao and Zhimin Shen conceived and designed the study. Lei Gao, Zhimin Shen, Hui Xu, and Fei Luo drafted this protocol. Peipei Zhang, Tianci Chai, and Sui Chen will perform the search, screening and extraction. Sui Chen and Mingqiang Kang have strictly reviewed this protocol and approved of publication.
